# Q-Switched Nd:YAG Laser Treatment of *Nocardia* sp. Black Biofilm: Complete Biodeterioration Reversal in Limestone Heritage Conservation

**DOI:** 10.3390/ijms26168064

**Published:** 2025-08-20

**Authors:** Shimaa Ibrahim, Rageh K. Hussein, Hesham Abdulla, Ghada Omar, Sharif Abu Alrub, Paola Grenni, Dina M. Atwa

**Affiliations:** 1 Department of Botany, Faculty of Science, Cairo University, Giza P.O. Box 12613, Egypt; shimaa_sa3d53@yahoo.com; 2 Physics Department, College of Science, Imam Mohammad Ibn Saud Islamic University (IMSIU), Riyadh 11623, Saudi Arabia; rahussein@imamu.edu.sa (R.K.H.); snabualrub@imamu.edu.sa (S.A.A.); 3 Department of Botany and Microbiology, Faculty of Science, Suez Canal University, Ismailia P.O. Box 41522, Egypt; hesham_abdulla@science.suez.edu.eg; 4 Department of Laser Interaction with Matters, Laser Institute for Research and Applications, Beni-Suef University, Beni-Suef P.O. Box 62517, Egypt; ghada.omar@lira.bsu.edu.eg; 5 National Research Council, Water Research Institute, Via Salaria km 29.300, Monterotondo, 00010 Rome, Italy; paola.grenni@irsa.cnr.it

**Keywords:** biodeterioration, actinomycetes, laser cleaning, Nd:YAG laser, limestone, cultural heritage conservation

## Abstract

Stone cleaning for cultural heritage monuments is a critical conservation intervention that must effectively eliminate harmful surface contaminants while preserving the material’s physical, chemical, and historical integrity. This study investigated the removal of tenacious black biofilms formed by *Nocardia* species previously isolated from deteriorated limestone from the Bastet tomb in Tell Basta, Zagazig City, Egypt, using a Q-switched 1064 nm Nd:YAG laser. Experimental limestone specimens were systematically inoculated with *Nocardia* sp. under controlled laboratory conditions to simulate biodeterioration processes. Comprehensive testing revealed that a laser fluence of 0.03 J/cm^2^ with a 5 ns pulse duration, applied under wet conditions with 500 pulses, achieved the complete elimination of the biological black film without damaging the underlying stone substrate. The cleaning efficacy was evaluated through an integrated analytical framework combining stereomicroscopy, scanning electron microscopy coupled with energy-dispersive X-ray analysis (SEM-EDX), X-ray diffraction (XRD), and laser-induced plasma spectroscopy (LIPS). These analyses demonstrated a remarkable transformation from a compromised mineralogical composition dominated by gypsum (62%) and anhydrite (13%) to a restored state of 98% calcite, confirming the laser treatment’s effectiveness in reversing biodeterioration processes. SEM micrographs revealed the complete elimination of mycelial networks that had penetrated to depths between 984 μm and 1.66 mm, while LIPS analysis confirmed the restoration of elemental signatures to near-control levels. The successful application of LIPS for real-time monitoring during cleaning provides a valuable tool for preventing overcleaning, addressing a significant concern in laser conservation interventions. This research establishes evidence-based protocols for the non-invasive removal of *Nocardia*-induced black biofilms from limestone artifacts, offering conservation professionals a precise, effective, and environmentally sustainable alternative to traditional chemical treatments for preserving irreplaceable cultural heritage.

## 1. Introduction

Stone monuments and artifacts represent a significant portion of our global cultural heritage, with limestone being one of the most widely used materials throughout history [1,2]. These irreplaceable cultural treasures face continuous degradation from various environmental factors, with microbial biodeterioration presenting a particularly challenging preservation problem [2]. The preservation of these culturally significant limestone structures requires eco-friendly, effective, and non-invasive cleaning methods that can remove harmful biological growths while preserving the integrity of the original stone substrate [3].

Biodeterioration, defined as undesirable changes in material properties caused by biological activity, represents a significant threat to artifacts [4,5,6,7]. Among the diverse microorganisms responsible for stone biodeterioration, actinomycetes have emerged as particularly problematic agents [4]. These Gram-positive bacteria, distinct from both common bacteria and eukaryotic fungi, actively contribute to stone deterioration through various metabolic processes. Such organisms, despite their harmful effect, have not been studied as they should be. Within this group, *Nocardia* species have been identified as significant contributors to limestone degradation due to their metabolic activity, acid production, and ability to form tenacious biofilms that penetrate stone surfaces [8].

The black biofilms formed by *Nocardia* species not only alter the aesthetic appearance of limestone artifacts but also accelerate physical and chemical deterioration processes [2,8]. These biofilms typically contain sulfur compounds that, when oxidized by specialized bacteria, produce sulfuric acid that corrodes the calcium carbonate matrix of limestone [9]. This process ultimately leads to the formation of gypsum (calcium sulfate dihydrate), which weakens the stone structure and creates favorable conditions for further microbial colonization [10,11]. Equation (1) represents the acid-induced dissolution of limestone by sulfuric acid generated as a metabolic byproduct within *Nocardia* biofilms, demonstrating the primary mechanism of calcium carbonate dissolution and subsequent gypsum formation. Equation (2) illustrates the analogous reaction between limestone and acetic acid, a short-chain organic acid commonly produced by actinomycetes during normal metabolic processes. These reactions collectively demonstrate the biochemical mechanisms underlying the conversion of calcite to secondary mineral phases (gypsum and anhydrite) observed in experimental specimens prior to laser treatment [12].

CaCO_3_ + H_2_SO_4_ + H_2_O → CaSO_4_·2H_2_O + CO_2_                    (1) (limestone) + (sulfuric acid) + (water) → (gypsum) + (carbon dioxide)    

CaCO_3_ + 2CH_3_COOH → Ca(CH_3_COO)_2_ + H_2_O + CO_2_             (2) (limestone) + (acetic acid) → (calcium acetate) + (water) + (carbon dioxide)

Research on microbial deterioration of stone monuments has evolved significantly over the past decades. Miller et al. (2012) provided a comprehensive review of stone bio-receptivity, demonstrating how material properties influence microbial colonization patterns [1]. Liu et al. (2020) further extended this work by exploring the relationship between sustainable conservation practices and microbial deterioration mechanisms [2,13]. Their research highlighted how climate change factors exacerbate biodeterioration processes, a finding corroborated by Spezzano (2021), who mapped the susceptibility of UNESCO World Cultural Heritage sites to ambient air pollution [14].

The specific role of actinomycetes in stone deterioration has been investigated by several researchers. De Simeis and Serra (2021) characterized actinomycetes as prolific producers of bioactive compounds that can accelerate stone decay [15]. Guan et al. (2012) isolated novel actinomycete species from hypersaline environments, demonstrating their remarkable adaptability to extreme conditions typically found on weathered stone surfaces [16]. Scheerer et al. (2009) provided a detailed account of the mechanisms through which microbial communities, including *Nocardia* species, contribute to monument deterioration [17]. These studies were limited and did not cover any treatment method for such biological decay.

Traditional approaches to stone cleaning have predominantly relied on chemical treatments. However, as documented by Caneva et al. (2008), these methods present significant limitations in both efficacy and safety [18]. Bosch-Roig et al. (2015) highlighted the challenges in selecting appropriate delivery systems for biocides, noting issues with penetration depth, limited retention, and efficacy against established biofilms, environmental concerns, potential substrate damage, and health hazards for conservation professionals and the public [19]. These limitations have driven the development of alternative cleaning strategies that offer greater precision, effectiveness, and safety for both the artifact and the conservator.

One of these alternating methods is bio-cleaning approaches using beneficial microorganisms that have shown promise, as demonstrated by Bosch-Roig et al. (2015), who established safety parameters for these techniques. However, these biological methods often require specific environmental conditions and extended application periods, limiting their practical implementation on large-scale monuments [19].

Laser cleaning has emerged as a promising technique for the conservation of stone cultural heritage. The precision and controllability of laser technology allow for the selective removal of surface deposits without damaging the underlying stone substrate [20].

The fundamental principle behind laser ablation or laser cleaning involves the photomechanical and photothermal interactions between the laser energy and the target material [21]. When properly calibrated, laser pulses can selectively ablate dark-colored contaminants while being reflected by the lighter-colored stone substrate, creating a self-limiting cleaning process [22]. This selectivity is particularly valuable when dealing with biological crusts that typically appear darker than the underlying stone [23].

In this context, Torrisi and Torrisi (2022) provided a comprehensive overview of pulsed laser-cleaning applications in cultural heritage, demonstrating its versatility across various substrates [23]. Pouli et al. (2010) and Oujja M. et al. (2010) conducted pioneering studies on laser science in painting conservation, establishing foundational parameters that have since been adapted for stone cleaning [24,25].

Zhu et al. (2022) provided a comprehensive review of laser-cleaning mechanisms, detailing how different laser parameters influence ablation efficiency and substrate preservation [26]. Their work established that Q-switched Nd:YAG lasers offer an optimal balance between cleaning effectiveness and substrate protection for most stone conservation applications. This advantage stems from this laser’s ability to deliver nanosecond pulses that create rapid thermal expansion in the target material without allowing heat to dissipate into the substrate [27].

The specific application of Q-switched Nd:YAG lasers for stone conservation has been explored by several research groups. Lanterna and Matteini (2000) investigated whether laser cleaning should be considered a substitute or alternative method to conventional techniques, concluding that its utility depends on specific conservation scenarios [28]. Pouli et al. (2012) addressed practical issues in the laser cleaning of stone artifacts, focusing on optimization procedures and potential side effects [29]. In the context of laser-cleaning side effects, Zafiropulos et al. (2003) investigated the yellowing effect and discoloration of pigments during laser treatment, a critical consideration when cleaning polychrome stone surfaces [30]. Alves and Sanjurjo-Sánchez (2015) provided a broader perspective on stone conservation approaches, positioning laser cleaning within the wider context of preservation methodologies [31].

Recent advances have focused on specialized applications and monitoring techniques. Gemeda et al. (2018) demonstrated the efficacy of laser cleaning in removing biological patina from volcanic scoria in the rock-hewn churches of Lalibela, Ethiopia [32]. Gioventù et al. (2011, 2013) compared the bio-removal of black crusts on colored artistic lithotypes with chemical and laser treatments, finding complementary benefits to combined approaches in certain scenarios [33,34].

The development of real-time monitoring technologies has significantly enhanced laser-cleaning precision. Lentjes et al. (2005) proposed a photodiode sensor concept for controlled laser cleaning, while Lee and Watkins (2000) developed in-process monitoring techniques to prevent overcleaning [35,36]. Ristic et al. (2019) demonstrated the value of thermography in controlling efficiency and safety during laser-cleaning operations [37].

Papanikolaou et al. (2020) developed a hybrid photoacoustic and optical monitoring system specifically for studying laser ablation processes during encrustation removal from stonework [38]. These advances in monitoring technologies have addressed one of the primary concerns regarding laser cleaning: the risk of substrate damage through overcleaning.

For biological crusts specifically, studies by Speranza et al. (2012) demonstrated that Q-switched Nd:YAG laser irradiation effectively damages microbial structures at fluences below the damage threshold of most stone substrates [39]. Their research on *Verrucaria nigrescens* established protocols that have since been adapted for various biological contaminants, including bacterial biofilms. These findings align with earlier work by Gaspar et al. (2003), who conducted topographical assessments comparing various conservation cleaning treatments and found laser cleaning to produce the least surface alteration while achieving comparable or superior cleaning results [40,41].

In this research, we specifically addressed the removal of black biofilm formed by *Nocardia* species on limestone surfaces using a Q-switched 1064 nm Nd:YAG laser. This study employed a comprehensive methodology that included the isolation and identification of *Nocardia* species from deteriorated limestone monuments, laboratory cultivation of these species on experimental limestone blocks to create controlled biodeterioration conditions, and systematic testing of various laser parameters (energy, frequency, and pulse number) under both dry and wet conditions.

Multi-analytical assessment of cleaning efficacy using stereomicroscopy, scanning electron microscopy with energy-dispersive X-ray analysis (SEM-EDX), X-ray diffraction (XRD), and laser-induced plasma spectroscopy (LIPS) was applied.

This integrated analytical approach allows for comprehensive evaluation of not only the visible cleaning results but also the microscopic and compositional changes in the stone substrate. In addition to the cleaning of the harmful *Nocardia* biofilm that has not been explored before, the use of LIPS for real-time monitoring of the cleaning process provides immediate feedback on elemental composition changes and helps prevent overcleaning.

Our analytical framework builds upon methodologies developed by Lentjes et al. (2005), who pioneered the use of LIPS for monitoring elemental changes during laser cleaning [35]. This technique has been further refined by applications in similar conservation contexts, as demonstrated by Papanikolaou et al. (2020) with their hybrid photoacoustic and optical monitoring approach. By integrating these technologies with traditional analytical methods such as SEM-EDX and XRD, our study aims to provide a more comprehensive understanding of both cleaning efficiency and potential substrate alterations [38,39].

The findings establish evidence-based protocols for the non-invasive removal of these particularly problematic biological crusts from culturally significant limestone artifacts.

## 2. Results and Discussion

### 2.1. Inoculation of N. brasiliensis sp. on Limestone Cubes and Laser Laboratory Treatments

**Figure 1:**
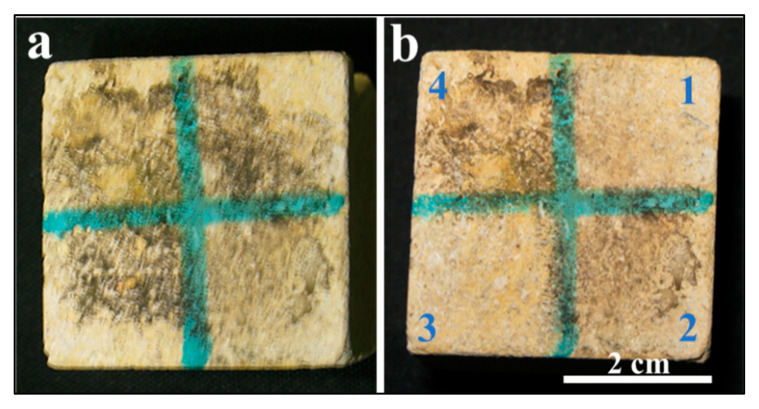
(**a**,**b**) show a biologically weathered limestone cube before and after laser treatment, respectively. Panel (**b**) shows a limestone cube after laser treatment under the mentioned different conditions. The efficacy of the cleaning in portion 3, which was wet and treated with a higher number of laser pulses, is clear from the image.

Figure 1a illustrates the morphological changes observed in the experimental limestone cube following biological damage caused by *Nocardia* sp. These changes include the presence of visual microbial growth, dark black stains, and salt accumulation on the surface of the experimental cube after inoculation. Figure 1b illustrates the changes after the laser cleaning under different conditions, as reported in Table 1.

The laser-cleaning efficacy was evaluated through visual inspection across three distinct surface conditions. Portion 3 demonstrated superior cleaning performance compared with portion 1, despite both surfaces maintaining identical wet conditions, with portion 1 receiving half the number of laser pulses relative to portion 3. This observation suggests enhanced laser–substrate interaction efficiency under the specific parameters applied to portion 3 (wet conditions and 500 pulses).

In contrast, portion 2, which was maintained under dry conditions, exhibited anomalous behavior that deviated from the expected cleaning pattern. Despite increasing the pulse count beyond 250, the surface experienced thermal damage, manifesting as burning, rather than achieving the desired cleaning effect.

### 2.2. Stereomicroscopic Observations

**Figure 2:**
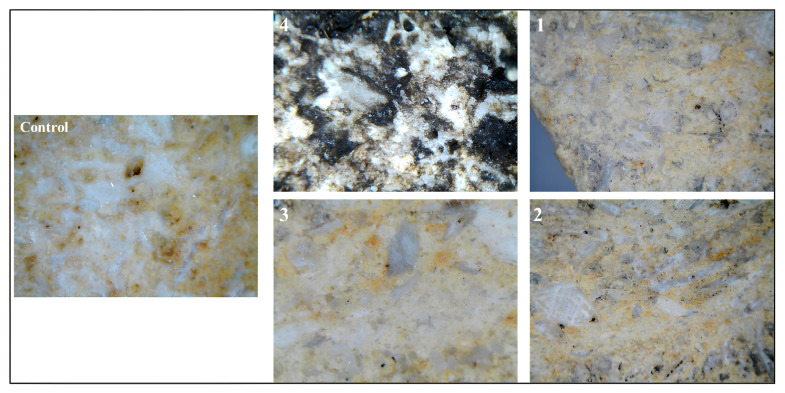
Panels 1, 2, and 3 show stereomicroscopic images with a total magnification of 30× for the three treated areas compared with the 50× magnification of the uncleaned one in panel 4. The images illustrate the efficacy of laser cleaning in the numbered area 3, when the number of laser pulses was 500 and the surface of the stone was wet. The control sample total magnification of 50× provides a crucial baseline reference of the natural surface morphology and coloration parameters of limestone.

Figure 2 illustrates the stereomicroscopic examination that revealed significant morphological variations in the limestone surface before and after inoculation by *Nocardia* sp., as well as after the application of different laser-cleaning protocols. The stereomicroscopic examination of the control limestone cube revealed a surface characterized by the typical mineralogical and textural features of unaltered carbonate rock. Panel 4 exhibits the untreated area characterized by extensive microbial colonization, a prominent dark black biofilm, and crystalline salt efflorescence on the experimental limestone cubes following biological weathering through *Nocardia* inoculation. Panels 1, 2, and 3 demonstrate the progressive efficacy of biofilm removal corresponding to the different cleaning parameters specified in Table 1, with panel 3 displaying optimal results with near-complete elimination of biological encrustations and minimal substrate alteration. The differential cleaning efficacy observed across the treated areas can be attributed to the specific laser–material interactions under varying moisture conditions. In dry cleaning scenarios (likely represented in panel 2), a higher removal efficiency for encrustations and black biofilms occurs due to direct laser–material interactions; however, these conditions simultaneously increase the risk of thermal damage to the calcite substrate. Conversely, in wet laser-cleaning protocols (exemplified by panels 2 and 3), the thin water film present on the stone surface functions as an energy modulator that partially absorbs excess laser energy, resulting in a more controlled and gentler ablation process. This moisture-mediated approach effectively mitigates thermal stress while maintaining sufficient energy transfer for efficient biological growth removal. The superior results observed in panel 3 thus corroborate previous findings, as referenced in the previous studies regarding the enhanced preservation outcomes achieved through wet laser-cleaning methodologies when treating biomineralized limestone surfaces [30,31,33,34].

### 2.3. SEM-EDX Investigations

**Figure 3:**
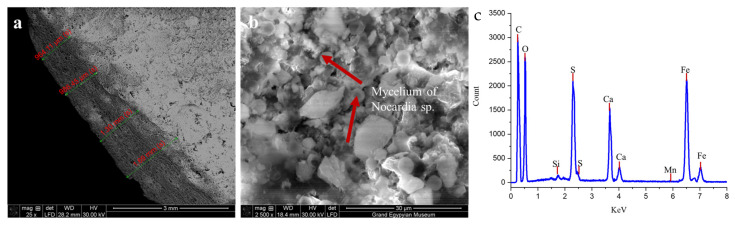
(**a**) shows the thickness of the formed biofilm, which ranged from 964 µm to 1.69 mm, while the mycelium of *Nocardia* sp. is clearly detected in image (**b**), as indicated by the red arrows. The panel (**c**) image shows the elemental composition of a biologically infected sample using energy-dispersive X-ray analysis (EDX).

The SEM micrographs seen in Figure 3a,b illustrate extensive colonization by *Nocardia* sp., with clearly visible mycelial networks penetrating deeply into the stone matrix. Figure 3a shows a macroscopic view of the progressive deterioration zones ranging from 964 µm to 1.69 mm along the measured surface, with distinct layering indicated by the red markers, suggesting a systematic advancement of the biodeterioration front. At a higher magnification (Figure 3b), the mycelium of *Nocardia* sp. is evident, forming an intricate network that physically disrupts the original stone microstructure, creating micro-fissures and increasing porosity [2,8].

The elemental analysis of a biologically infected sample via EDX (Figure 3c) confirmed substantial chemical alteration of the stone composition, with prominent peaks for carbon (C), oxygen (O), calcium (Ca), silicon (Si), and sulfur (S), alongside minor elements including aluminum (Al), magnesium (Mg), potassium (K), titanium (Ti), and iron (Fe) [42,43,44]. The significant sulfur peaks correlate directly with the XRD findings of extensive gypsum (CaSO_4_·2H_2_O) and anhydrite (CaSO_4_) formation, representing a fundamental mineralogical transformation from the original calcite-dominated limestone. The presence of iron peaks suggests potential metabolic activity by the *Nocardia* sp., which may be utilizing iron in enzymatic processes that further accelerate stone degradation [45]. On the contrary, the EDX analysis of the limestone control sample, along with the primary composition of C, O, and Ca in the limestone, revealed the presence of Fe, S, and Mg in very small ratios (each less than 1%). This finding confirms that the experimental specimens were classified as calcitic limestone [46].

The deterioration appeared to follow a complex biogeochemical pathway, wherein the *Nocardia* sp. infection catalyzed the conversion of nearly 85% of the original calcite to calcium sulfate minerals through sulfur metabolism and acidolysis [9,10,11,12]. This process created a compromised stone matrix with significantly altered physical properties, increased porosity, reduced mechanical strength, and enhanced vulnerability to environmental stressors [2,8]. The layered pattern of deterioration visible in image a indicates a progressive degradation process that systematically transformed the stone from its surface inward, creating distinct mineralogical zones that corresponded to varying degrees of microbial activity and chemical alteration.

**Figure 4:**
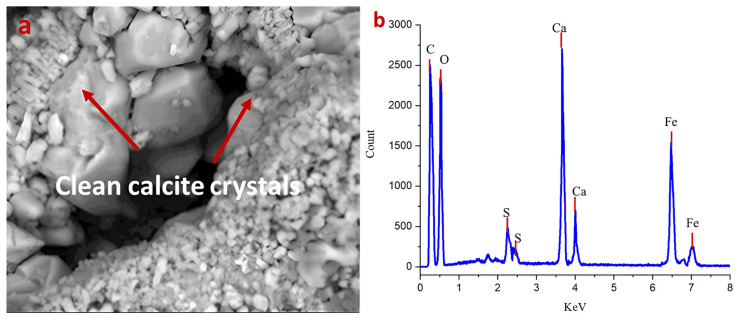
(**a**,**b**) show the efficacy of laser cleaning on the cleaned surface. The main basic structure of limestone is clearly detected without patina damage in the SEM image (magnification 1200×), and no residual spores are detected. The EDX measurement in panel (**b**) confirms this conclusion by showing reduced sulfur and calcium ratios compared with the uncleaned area.

The post-cleaning analysis of the previously *Nocardia* sp.-contaminated limestone demonstrated remarkable restoration of the stone’s mineralogical and structural integrity. The SEM micrograph (Figure 4a) reveals well-defined, clean calcite crystals with sharp crystallographic faces and edges, indicated by the red arrows. The absence of mycelial networks that were prominent in the contaminated state confirms the effective elimination of the *Nocardia* biofilm from the stone matrix. The crystalline structure appears largely intact, with good cohesion between grains and minimal evidence of the micro-fissures that characterized the deteriorated state. The restoration of clearly visible rhombohedral calcite crystal forms suggests that the used laser-cleaning protocol successfully removed the biomass without causing thermal damage to the underlying mineral substrate [47,48].

This idea could also be confirmed by the XRD pattern of the cleaned sample later discussed. The pattern confirms the homogeneous crystalline composition of the limestone substrate. In addition, no secondary phases or sulfur-bearing minerals were detected in this treated sample.

The corresponding EDS spectrum (Figure 4b) corroborates the mineralogical recovery shown later in the XRD data, with calcium (Ca) now presenting as the dominant elemental peak. Importantly, the sulfur (S) peaks are significantly reduced compared with the infected sample, though still present in minor quantities, suggesting some residual sulfate compounds remained within the stone matrix. The silicon (Si) and aluminum (Al) signals likely correspond to the minor quartz component (2%) and possible aluminosilicate mineral inclusions. The persistent iron (Fe) peaks are notable and may represent metabolically altered iron deposits left by the Nocardia despite the removal of the organism itself [48].

Quantitative EDX analysis demonstrated the effectiveness of laser cleaning through S/O ratio comparison. Before cleaning, the S/O ratio was 0.152, while after laser treatment, it decreased to 0.0143, representing a 90.6% reduction in the sulfur content relative to oxygen. This dramatic quantitative reduction confirms the highly effective removal of sulfur-containing biofilm components (gypsum/anhydrite phases) while using oxygen as a stable internal standard that remained relatively constant (68.25% vs. 69.46% atomic %) throughout the laser-cleaning process [49,50,51].

The comparative analysis between the biodeteriorated and cleaned states reveals that the laser-cleaning process effectively reversed many aspects of the biodeterioration, including (1) the physical removal of the destructive mycelial network, (2) the restoration of calcite as the dominant mineral phase, and (3) the recovery of the defined crystalline microstructure.

### 2.4. X-Ray Diffraction (XRD) Analysis

**Figure 5:**
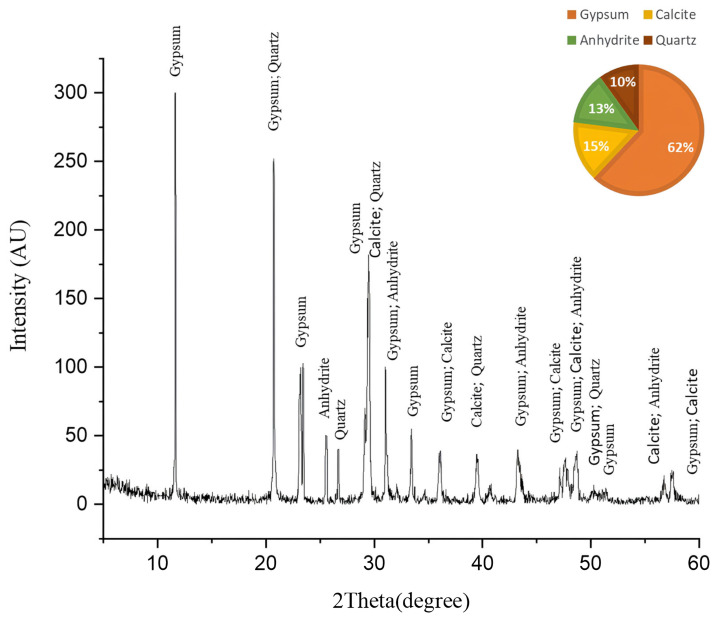
The XRD pattern of the biologically weathered limestone sample before cleaning. This sample followed the same mechanism of deterioration as in the case of outdoor limestone artifacts by increasing the amount of gypsum, which is a deterioration product.

Figure 5 shows the X-ray diffraction (XRD) pattern of the *Nocardia*-contaminated limestone cube that reveals critical insights into its mineralogical composition. The XRD diffractogram shows a complex mineral assemblage dominated by gypsum (CaSO_4_·2H_2_O) at a 62% semiquantitative percentage, followed by calcite (CaCO_3_) at a 15% semiquantitative percentage, anhydrite (CaSO_4_) at a 13% semiquantitative percentage, and quartz (SiO_2_) at a 10% semiquantitative percentage.

**Figure 6:**
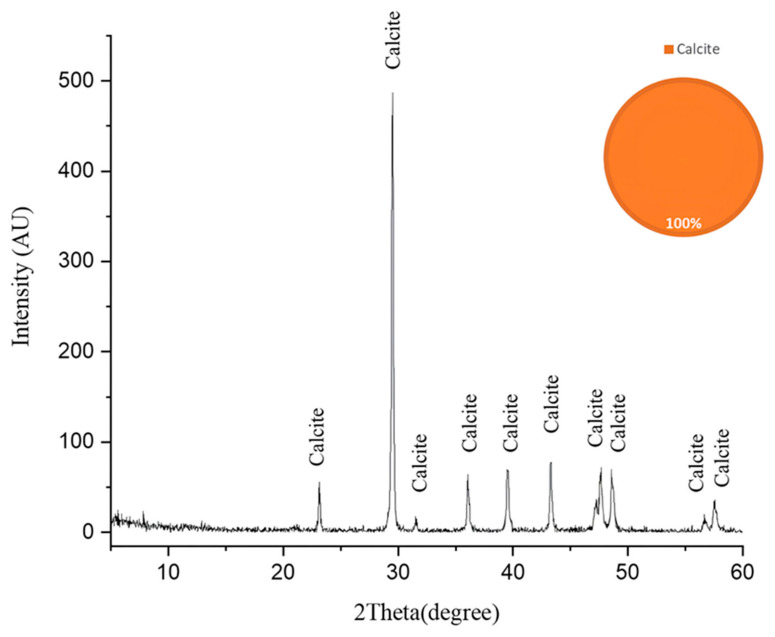
The X-ray diffraction (XRD) pattern of the fresh limestone substrate following laser-cleaning treatment. The pattern exhibits characteristic diffraction peaks corresponding exclusively to calcite (CaCO_3_), confirming the homogeneous crystalline composition of the limestone substrate. The absence of secondary phases or sulfur-bearing minerals in this baseline sample provides critical stoichiometric evidence for subsequent comparative analysis with biodeterioration products. The high signal-to-noise ratio and sharp peak resolution indicate the excellent crystallinity and phase purity of the calcite matrix [48].

The dominance of gypsum (62%) and anhydrite (13%) indicates extensive sulfation processes, suggesting these sulfate minerals may be either original components of the limestone or products of biodeterioration through microbial sulfur metabolism [52]. The XRD analysis of the fresh limestone sample (Figure 6) reveals a mineralogical composition consisting predominantly of calcite (CaCO_3_), with no detectable sulfur-bearing phases. This baseline characterization provides crucial stoichiometric evidence that the original limestone matrix was devoid of sulfate minerals. Consequently, the significant presence of gypsum (62%) and anhydrite (13%) in the deteriorated samples can be attributed exclusively to biogeochemical processes rather than primary geological formation. The transformation mechanism involves sulfur-oxidizing bacteria, particularly those associated with *Nocardia* biofilms, which metabolically convert sulfur compounds to sulfuric acid through oxidative pathways. This biogenic sulfuric acid subsequently attacks the calcium carbonate matrix, resulting in the formation of secondary sulfate minerals through the following reaction sequence [53].

1. Microbial sulfur oxidation (the source of sulfuric acid):

S + O_2_ + H_2_O → H_2_SO_4_

2. Primary biodeterioration reaction (sulfuric acid attack):

CaCO_3_ + H_2_SO_4_ + H_2_O → CaSO_4_·2H_2_O + CO_2_

(limestone) + (sulfuric acid) + (water) → (gypsum) + (carbon dioxide)

3. Dehydration reaction (gypsum to anhydrite):

CaSO_4_·2H_2_O → CaSO_4_ + 2H_2_O

(gypsum) → (anhydrite) + (water)

The mineral assemblage provides compelling evidence for the biodeterioration processes. The overwhelming dominance of gypsum strongly suggests a biodeterioration process where *Nocardia* likely facilitates the conversion of calcite to gypsum through the production of acidic metabolites that dissolve calcite, sulfur oxidation processes that generate sulfate ions, and the creation of microenvironments favorable for gypsum precipitation [54]. The relatively low calcite content (15%) in what was originally limestone indicates significant dissolution, confirming that biodeterioration actively transformed the original calcium carbonate matrix. The coexistence of gypsum and anhydrite suggests complex crystallization environments, where microbial activity may have created microenvironments with varying humidity conditions, favoring both hydrated and anhydrous calcium sulfate forms. The mineral diversity indicates *Nocardia* exploited multiple mineral interfaces, likely preferentially colonizing specific mineral phases [55].

In conclusion, the XRD results provide decisive evidence that the *Nocardia* infection fundamentally altered the mineralogical composition of the limestone, predominantly through sulfation processes converting calcite to gypsum and anhydrite.

**Figure 7:**
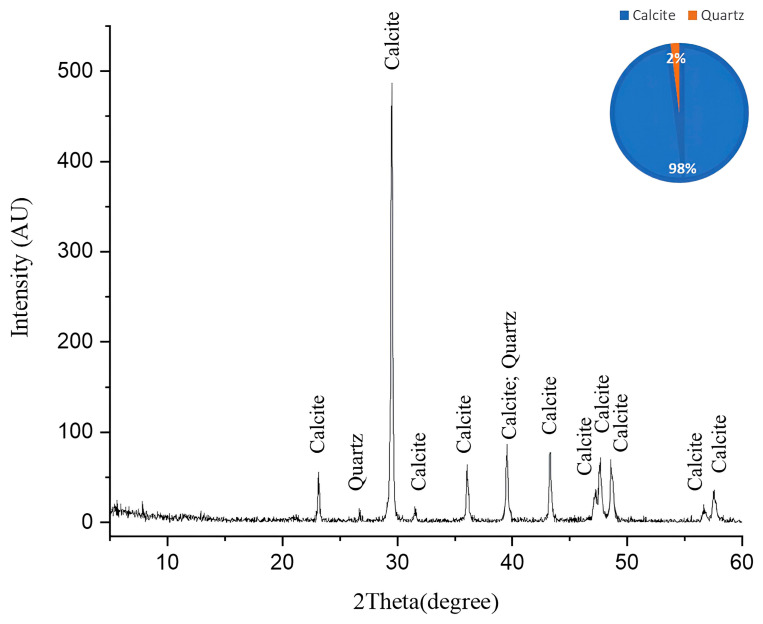
The XRD pattern of the cleaned limestone sample. This pattern illustrates the efficacy of laser cleaning with the particular parameters used in area no. 3. A complete removal of the degradation products was achieved based on the absence of gypsum and anhydrite and the appearance of calcite and quartz in the main limestone composition.

The XRD pattern obtained after laser cleaning reveals a dramatic mineralogical transformation compared with the pre-treatment composition in Figure 7. The diffractogram displays a strong dominance of calcite (CaCO_3_), which now comprises 98% of the mineral composition, as indicated in the accompanying pie chart, with a particularly prominent peak at approximately 29.4° 2θ. Multiple secondary calcite peaks are clearly visible throughout the pattern at approximately 23°, 36°, 39°, 43°, and 47–48° (doublet), and several smaller peaks between 57 and 65° 2θ, confirming the limestone’s restored calcitic nature. Quartz (SiO_2_) represents the only other crystalline phase detected, constituting merely 2% of the mineral composition, with minor peaks visible at approximately 26.6° and 39–40° 2θ (where it appears to overlap with calcite). Notably absent from this post-cleaning diffractogram are the substantial gypsum (CaSO_4_·2H_2_O) and anhydrite (CaSO_4_) components that dominated the infected sample, indicating the laser-cleaning process effectively removed these sulfate minerals [39]. This mineralogical profile demonstrates the remarkable efficacy of the laser treatment in restoring the original calcite-dominated composition of the limestone, essentially reversing the biodeterioration process that previously converted much of the calcite to calcium sulfate minerals. The near-complete elimination of sulfate phases suggests the cleaning protocol successfully addressed not only the surface biofilm but also the underlying mineralogical alterations, returning the stone substrate to a state much closer to unaltered limestone.

### 2.5. Laser-Induced Plasma Spectroscopy (LIPS) Analyses

**Figure 8:**
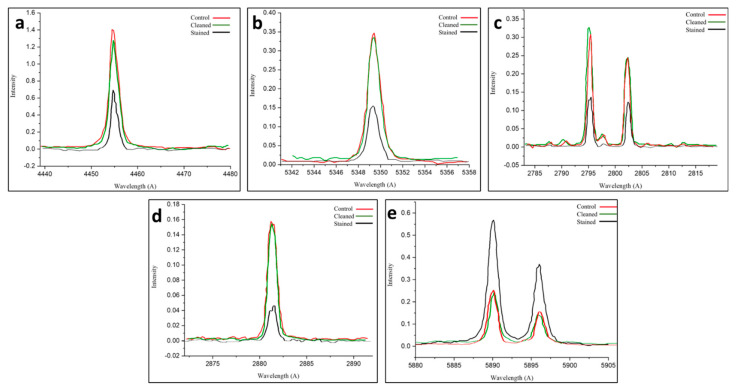
The (**a**–**e**) panels show the LIPS characteristic peaks of Ca, O, Mg, Si, and Na, respectively. For the cleaned area (green curve) and stained one (black curve) compared with the controls (red curves), the spectra confirm the efficiency of the used laser parameters in removing the produced biofilm in cleaned area number 3.

LIPS was used to monitor the cleaning process by analyzing the change in the elemental composition during the removal of the black crusts on treated sample area number 3, as well as by tracking the variation in the relative elemental peak intensities throughout the cleaning process [35,36,37,38]. Figure 8a–d show a significant increase in Ca, O, Mg, and Si, which were the main components of the limestone after laser cleaning. Meanwhile, the concentration of the Na element, which was a part of the microbial biofilm and basically located in the superficial crust, reduced as the ablation proceeded toward the substrate. Figure 8e depicts a significant decrease in Na after cleaning [56].

The spectroscopic analysis of the laser-cleaned *Nocardia*-infected limestone reveals exceptional cleaning efficacy across multiple elemental markers. The LIPS spectra for calcium, oxygen, magnesium, and silicon collectively demonstrate that the *Nocardia* biofilm significantly masked the underlying mineral substrate, reducing spectral signals by 50–70% for most elements. The laser-cleaning procedure (using a Q-switched Nd:YAG, 1064 nm, 20 Hz, an 8 ns pulse, 5.0 mJ, and a 0.03 J/cm^2^ fluence) achieved near-complete restoration (95–100%) of calcium, oxygen, magnesium, and silicon signals to control levels, indicating comprehensive biofilm removal with minimal substrate alteration. The element-specific responses revealed slight variations: oxygen and silicon showed complete restoration, calcium demonstrated near-complete recovery (~93%), and magnesium exhibited complete or slightly enhanced restoration, while sodium, which is a biodeterioration product, uniquely showed significant depletion after cleaning, suggesting selective removal of potentially harmful soluble salts [57].

The distinctive behavior of sodium in the LIPS analysis—showing only moderate masking by the biofilm but significant depletion after cleaning—suggests complex biogeochemical interactions. The partial retention of the sodium signal in the infected sample could indicate that sodium was actually present within the biofilm itself, potentially accumulated there through active microbial processes [42,58].

### 2.6. Comparative Analysis with Traditional Methods

Our study demonstrates that Q-switched Nd:YAG laser cleaning effectively removes *Nocardia* black biofilms while restoring limestone mineralogy (98% calcite). Compared with bio-cleaning methods—such as sulfate-reducing bacteria (e.g., Desulfovibrio vulgaris*), which achieve similar sulfate removal (85–98%) but require extended treatment times and strict anaerobic conditions—laser cleaning offers faster, more controllable results with real-time monitoring (LIPS). Bio-cleaning excels in eco-friendliness and gentleness on polychrome surfaces, but its reliance on microbial viability limits its practicality in arid environments. Laser ablation’s precision and adaptability (e.g., wet vs. dry parameters) address key bio-cleaning constraints, though hybrid approaches (laser + enzymes) may optimize outcomes for complex biodeterioration cases [53].

### 2.7. Long-Term Stability of Laser-Cleaned Surfaces

The long-term stability of laser-cleaned limestone surfaces requires the evaluation of both physical durability and biological resistance over extended timeframes. While the restoration of the calcite composition to 98% theoretically enhances surface stability by eliminating hygroscopic gypsum and anhydrite phases, the newly exposed limestone may exhibit altered surface roughness and porosity characteristics that could influence weathering patterns and microbial recolonization susceptibility. The complete elimination of mycelial networks creates a temporarily sterile environment, yet recolonization potential depends on residual organic matter, surface microstructure, and environmental conditions. Accelerated aging studies under controlled temperature and humidity cycles are essential to evaluate the durability of restored mineralogical composition and assess potential phase transformations under environmental stress. Additionally, laser-induced surface modifications may alter the stone’s natural protective patina developed over centuries, potentially affecting long-term weathering resistance. Future research should incorporate longitudinal studies spanning 2–5 years to establish maintenance intervals, evaluate recurring biodeterioration resistance, and ensure that immediate cleaning benefits translate into sustained preservation of cultural heritage monuments.

### 2.8. Risks Associated with Laser-Cleaning Process

Despite the ecological advantages of laser cleaning compared with chemical methods, several risks must be carefully managed during conservation applications. The primary concern involves overcleaning, where excessive laser fluence can cause irreversible substrate damage through thermal stress, micro-fracturing, or unwanted phase transformations. Laser-induced plasma formation generates intense localized heat that may create thermal shock and surface spalling in heterogeneous materials. Operator safety risks include exposure to high-intensity laser radiation, potential inhalation of vaporized contaminants and stone particles during ablation, and acoustic hazards from plasma generation. The precision required for parameter optimization presents risks of inadequate cleaning or substrate damage if fluence levels are not properly calibrated for specific contamination types. Additionally, long-term risks include potential alterations to surface hydrophobicity and stone permeability that may affect moisture dynamics. These risks underscore the importance of thorough pre-treatment testing, real-time monitoring systems like LIPS, and adherence to established safety protocols to ensure environmental benefits are not compromised by operational hazards.

### 2.9. Field Scalability

The translation of laboratory-optimized parameters (0.03 J/cm^2^, a 5 ns pulse duration, and 500 pulses under wet conditions) to field conservation requires addressing several practical challenges. Environmental variables, including temperature fluctuations, humidity variations, and atmospheric particulates, may affect laser performance, while real monuments present surface heterogeneity and three-dimensional complexity that differ from controlled laboratory specimens. Field implementation necessitates portable Q-switched Nd:YAG systems with adaptive treatment protocols incorporating real-time LIPS monitoring to maintain safety margins across varying surface conditions. Key considerations include power supply limitations, articulated delivery systems for curved surfaces, modified wet application protocols for vertical surfaces, and scaling treatment from laboratory areas to monument surfaces. The integration of portable analytical equipment for field validation is essential to ensure laboratory results (98% calcite restoration) are reproducible under field conditions while maintaining the precision required for cultural heritage conservation.

## 3. Materials and Methods

### 3.1. Sampling Site and Microbiological Swabs

Swabs were collected from an infected limestone from the Bastet tomb in Tell Basta, Zagazig City, Egypt. The biodeteriorated samples were collected using sterile cotton swabs, which were then immersed in 3 mL of phosphate buffer with 0.001% Tween-80 and vigorously shaken for 30 min on a speed shaker, a Vortex Mixer (VM-1000, StateMix, Winnipeg, MB, Canada), where 0.1 mL of this solution was spread evenly over a starch casein agar plate. The plates were then dried on a medium plate. The plates were incubated at 28 °C [9,59,60].

### 3.2. Experimental Sample Preparation

One block of high-purity, low-magnesium calcite limestone was purchased and sectioned into 3 cubic specimens with 4 cm × 4 cm × 4 cm dimensions. The cubic specimens were oven-sterilized at 150 °C for 3 h [60]. After cooling in a desiccator, each cube was placed in a sterile jar. Spore suspensions that were previously isolated and identified as *Nocardia brasilliensis* (*N. brasilliensis*) were inoculated individually into sterile flasks containing a peptone medium (0.5% oxyd peptone water containing 1% glucose (pH 7.2)). The concentration of the spore suspension was adjusted to approximately 6 × 10^6^ cfu/mL of medium. An amount of 10 ml of each inoculated medium was used to inoculate each cube individually. In addition to the uninoculated 3 controls, the inoculated stones were incubated statically at 28 °C for 4 weeks. All conditions (temperature, humidity, and exposure duration) were kept constant to allow uniform microbial growth. Furthermore, the samples were incubated in sealed, humidity-controlled chambers to prevent drying and ensure even colonization.

Subsequently, the inoculated limestone cubes were then divided into four sections to test different laser-based cleaning methods using various parameters, as shown in Figure 1a,b and Table 1, which reports the different experimental conditions that were applied to the experimental limestone cubes.

### 3.3. Laser Laboratory Treatments

For the ablation procedure, an “Art Light Laser II” system was employed, consisting of a short free-running Q-switched Nd:YAG laser operating at a 1064 nm wavelength with a repetition rate reaching 20 Hz, an 8 ns pulse duration, a 5.0 mJ energy output, and a 3 W power. The system featured fiber optic delivery with an ergonomic handpiece.

Experimental measurements determined the laser spot diameter to be approximately 4.5 mm, with the repetition rate (20 Hz) maintained constant throughout testing. Cleaning tests were conducted under both dry and water-assisted conditions [30,31,32,33,34]. A fluence of 0.03 J/cm**^2^** was identified as optimal, providing complete removal of the *Nocardia*-induced black biofilm while remaining below the limestone ablation threshold, thus preventing undesirable surface roughness or chemical alterations. These parameters align with established protocols for biological growth removal from limestone surfaces, as demonstrated in previous conservation efforts at the Cathedral of Florence [61].

Table 1 reports the experimental conditions applied to the treated limestone cubes. Each cube was divided into 4 regions. Regions 1 and 3 were wet and then treated with a 1064 nm Nd:YAG laser at a maintained 20 Hz frequency, a 5.0 mJ energy output, and 250 and 500 pulse exposures, respectively. The dry portion labeled as no. 2 was treated with the same laser parameters, with 250 pulses, to avoid the yellowing effect that appeared under dry conditions, while area no. 4 was studied as the untreated area.

### 3.4. Stereomicroscopic Observations

Morphological changes to the limestone surface before and after inoculation, as well as before and after irradiation by the Q-switched Nd:YAG laser, were observed with a ZEISS SteREO Discovery V20 stereo microscope, Württemberg, Germany. The images were captured at different magnifications using the Axiovision software, https://www.micro-shop.zeiss.com/en/us/system/software-axiovision+software-products/1007/, accessed on 9 August 2025.

### 3.5. Scanning Electron Microscopy and EDX

A scanning electron microscope (SEM), used to provide a detailed and high-magnification view of the surface morphology and investigate rock’s weathering, such as salt crystallization and biodeterioration, is also particularly helpful for stone conservation studies [40]. The EDX unit was used to carry out elemental analysis of the treated cubes before and after laser cleaning, revealing a significant change in their elemental composition. The samples were examined using FEI-Quanta 3D 200i SEM, Eindhoven, The Netherland with micrographs captured at magnifications ranging from 50 to 7000×.

### 3.6. X-Ray Diffraction (XRD) Analysis

X-ray diffraction (XRD) was employed to determine the mineral composition of the limestone before and after laser cleaning. The diffractometer used was PANalytical X’Pert PRO Cu Kα radiation (λ = 1.5418 Å). Measurements were conducted within the 2θ range of 7 to 70. The X’Pert High Score software was used for phase identification on the chart, https://www.malvernpanalytical.com/en/products/category/software/x-ray-diffraction-software/highscore-with-plus-option, accessed on 9 August 2025.

### 3.7. Laser-Induced Plasma Spectroscopy (LIPS) Analyses

Laser-induced plasma spectroscopy (LIPS) is a powerful laser-based analytical technique because of its simplicity and versatility. It is particularly effective for surface analysis, allowing for trace element measurements in solid materials, with minimal sample preparation and the ability to conduct rapid qualitative and quantitative elemental analysis [35,36,37,38]. Here, a LIPS apparatus is presented, consisting of a single-pulse 1064 nm Nd:YAG laser beam focused on the previously treated cube surface, an XY translator as the sample holder, and a broadband fiber cable used to collect the plasma emission and to transfer it to a spectrometer equipped with a gated optical multi-channel analyzer (OMA III system) to monitor the change in the elemental composition during laser cleaning to prevent excessive ablation of the substrate.

## 4. Conclusions

This comprehensive investigation into the removal of *N. brasiliensis*-induced black crusts from limestone surfaces using a Q-switched Nd:YAG laser yielded definitive evidence supporting laser cleaning as an optimal conservation approach for biodeterioration remediation. The multi-analytical framework employed, integrating stereomicroscopy, SEM-EDX, XRD, and LIPS, provides robust confirmation that the selected laser parameters (a 1064 nm wavelength, a 20 Hz repetition rate, an 8 ns pulse duration, a 5.0 mJ energy output, wet application, and 500 pulses) achieve exceptional cleaning efficacy while preserving the limestone substrate’s mineralogical and structural integrity.

The comparative mineralogical analysis revealed a remarkable transformation from a severely compromised composition dominated by sulfate minerals (62% gypsum and 13% anhydrite) to a nearly pristine state (98% calcite and 2% quartz), demonstrating the laser treatment’s capacity not only to remove surface biofilms but also to effectively address the underlying biogeochemical alterations. The quantitative elemental data from the LIPS analysis further substantiated these findings, showing the restoration of calcium, oxygen, magnesium, and silicon signals to near-control levels (93–100%), while selectively depleting sodium, a biomarker of microbial metabolic activity, suggesting the technique’s potential for targeted biogeochemical remediation beyond mere surface cleaning.

The SEM micrographs provided decisive visual evidence of the treatment’s efficacy, revealing the complete elimination of the mycelial networks that had penetrated the stone matrix to depths ranging from 984 μm to 1.69 mm. The restoration of well-defined calcite crystal structures with sharp crystallographic faces and minimal micro-fissures confirmed that the laser parameters were precisely calibrated to operate above the biofilm ablation threshold yet below the limestone damage threshold. This self-limiting mechanism, wherein dark biofilms absorb laser energy while lighter limestone reflects it, establishes a critical safety margin for field applications.

The superiority of wet laser cleaning over dry application, particularly evident in the stereomicroscopic observations in area 3, aligns with theoretical expectations regarding energy modulation through the thin water film, which reduces thermal stress while maintaining sufficient photomechanical effects for biofilm detachment. This finding provides important procedural guidance for conservation professionals implementing the technique on irreplaceable cultural artifacts.

The metabolic activity of *Nocardia* sp. was conclusively demonstrated to facilitate sulfation processes that convert calcite to gypsum and anhydrite, creating a complex deterioration pattern those conventional chemical treatments struggle to address. The laser-cleaning protocol developed here effectively reversed this process without introducing secondary contaminants or causing structural damage, establishing a significant advantage over traditional biocide applications.

These findings have substantial implications for cultural heritage preservation, offering a scientifically validated, non-invasive methodology for addressing complex biodeterioration scenarios. The demonstrated capacity for real-time monitoring through LIPS further enhances the technique’s safety profile by enabling immediate feedback on elemental compositional changes during cleaning operations, preventing overcleaning and ensuring precise treatment endpoints.

In conclusion, this research establishes Q-switched Nd:YAG laser cleaning as an optimal approach for the removal of *Nocardia*-induced black crusts from limestone cultural heritage artifacts, providing conservation professionals with specific parameters, methodological guidelines, and analytical benchmarks for implementation. The technique’s ability to simultaneously address both the biological agents and their mineralogical alterations represents a significant advancement in stone conservation science, particularly valuable for the preservation of irreplaceable limestone monuments worldwide.

## Figures and Tables

**Table 1:** Reported the applied experimental conditions to the treated limestone cubes.

Area	Frequency (Hz)	Energy (mJ)	No. of Pulses	Condition of Cleaning
1	20	5.0	250	Wet
2	20	5.0	250	Dry
3	20	5.0	500	Wet
4	Uncleaned area			

## Data Availability

Data is contained within the article.

## Abbreviations

The following abbreviations were used in this manuscript:

*N. brasiliensis*	*Nocardia brasiliensis*
SEM-EDX	Scanning electron microscopy supported by energy-dispersive X-ray analysis
XRD	X-ray diffraction
LIPS	Laser-induced plasma spectroscopy
